# Translation regulation by RNA stem-loops can reduce gene expression noise

**DOI:** 10.1186/s12859-024-05939-8

**Published:** 2024-10-22

**Authors:** Candan Çelik, Pavol Bokes, Abhyudai Singh

**Affiliations:** 1 Department of Applied Mathematics and Statistics, Comenius University, 84248 Bratislava, Slovakia; 2 Department of Industrial Engineering, Istanbul Aydin University, 34295 Istanbul, Turkey; 3 Mathematical Institute, Slovak Academy of Sciences, 81473 Bratislava, Slovakia; 4 Department of Electrical and Computer Engineering, University of Delaware, Newark, 19716 USA

**Keywords:** Stochastic gene expression, Master equation, Stochastic simulation

## Abstract

**Background:**

Stochastic modelling plays a crucial role in comprehending the dynamics of intracellular events in various biochemical systems, including gene-expression models. Cell-to-cell variability arises from the stochasticity or noise in the levels of gene products such as messenger RNA (mRNA) and protein. The sources of noise can stem from different factors, including structural elements. Recent studies have revealed that the mRNA structure can be more intricate than previously assumed.

**Results:**

Here, we focus on the formation of stem-loops and present a reinterpretation of previous data, offering new insights. Our analysis demonstrates that stem-loops that restrict translation have the potential to reduce noise.

**Conclusions:**

In conclusion, we investigate a structured/generalised version of a stochastic gene-expression model, wherein mRNA molecules can be found in one of their finite number of different states and transition between them. By characterising and deriving non-trivial analytical expressions for the steady-state protein distribution, we provide two specific examples which can be readily obtained from the structured/generalised model, showcasing the model’s practical applicability.

## Background

Biochemical processes such as stochastic gene expression are inherently subject to random fluctuations that lead to noise in the number of constituents [1]. Quantifying the dynamics and the noise in such stochastic processes is an intense study of various research areas. Under simplest assumptions, gene expression is described as a two-step stochastic process comprised of transcription and translation that play a significant role in determining the levels of gene products. While RNA polymerase enzymes produce mRNA molecules in the former, protein synthesis takes place by ribosomes in the latter. Because of the similarity, it is often referred to as the (classical) two-stage gene-expression model. How gene-expression regulation affects the level of gene products such as mRNA and protein is a question of interest.

The contributions to gene expression noise give rise to cell-to-cell variability in the mRNA and protein levels [2–9]. The noise emerges from different sources, namely *intrinsic* and *extrinsic* noise [10, 11]; yet, structural elements such as stem-loops can also contribute to noise by binding to an untranslated region of mRNA [12, 13]. The untranslated regions of mRNAs often contain these stem-loops that can reversibly change configurations making individual mRNAs translationally active/inactive [14].

From a mathematical perspective, the dynamics of gene-expression mechanisms can be described in deterministic and stochastic settings by means of ordinary differential equations (ODEs) and Master equation formulation, respectively. On the other hand, hybrid models have also been proposed as a combination of the preceding two [15–17]. Only a few of those provide an explicit solution to the (classical) two-stage gene-expression model [18, 19]; most of the studies are based on Monte Carlo simulations, which are usually computationally expensive.

In recent decades, the (classical) two-stage model of gene expression has been extensively utilised to elucidate the underlying mechanisms of stochastic processes in living cells [19–22]. In particular, it has been extended by the regulation of transcription factors, which affect gene expression by modulating the binding rate of RNA polymerase [23]. Specifically, the stochastic dynamics of the classical two-stage model of gene expression is described by the reaction scheme [18, 24]

1 $$\begin{array}{*{20}{c}} {\phi \xrightarrow{{{\lambda ^m}}}m{\text{RNA}},}&{m{\text{RNA}}\xrightarrow{{{\gamma ^m}}}\phi ,}&{m{\text{RNA}}\xrightarrow{{{\lambda ^p}}}m{\text{RNA}} + {\text{protein}},}&{{\text{protein}}\xrightarrow{{{\gamma ^p}}}\phi ,} \end{array}$$

where $\lambda ^m$ is the mRNA production rate, $\lambda ^p$ is the protein translation rate, and $\gamma ^m$ and $\gamma ^p$ are the decay rate constants of mRNA and protein species, respectively. Here and henceforth, *m* and *p* in the superscript indicate the mRNA and protein species, respectively.

As a generalisation of the (classical) two-stage model, some studies in the literature consider a set of multiple gene states and investigate the dynamics of stochastic transitions among these states [25–28]. Here, we study a structuration/generalisation of the classical two-stage gene-expression model (1), which takes into account multiple mRNA states. More specifically, after being transcribed, mRNA molecules are considered to be transitioning among their different states at constant reaction rates. Subsequently, the nascent mRNA molecule is translated, and protein is degraded. The schematic of the reactions describing this system is given by the following set of chemical reactions:

2 $$\begin{gathered} \begin{array}{*{20}{c}} {\phi \xrightarrow{{\lambda _i^m}}m{\text{RN}}{{\text{A}}_i}\xrightarrow{{\gamma _i^m}}\phi ,}&{i = 1, \ldots ,K,} \\ {m{\text{RN}}{{\text{A}}_i}\xrightarrow{{{q_{ij}}}}m{\text{RN}}{{\text{A}}_j},}&{i,j = 1, \ldots ,K,\quad i \ne j,} \\ {m{\text{RN}}{{\text{A}}_i}\xrightarrow{{\lambda _i^p}}m{\text{RN}}{{\text{A}}_i} + {\text{protein}},}&{i = 1, \ldots ,K,} \end{array} \hfill \\ {\text{protein}}\xrightarrow{{{\gamma ^p}}}\phi ,{\text{ }} \hfill \\ \end{gathered}$$

where $\lambda _i^m$ and $\gamma _i^m$ are the production and decay rates for an mRNA molecule in *i*-th state, respectively. The term $q_{ij}$, $i \ne j$, denotes the mRNA transition rate from state *i* to state *j*, $\lambda _i^p$ and $\gamma ^p$ are the protein translation and decay rates, respectively. The subscript is reserved for multiple mRNA states. All model parameters and their biological meaning are listed in Table 1.

**Table 1:** Model parameters and their biological meaning used in all model variations

Parameter	Meaning
$\lambda ^m$	mRNA production rate
$\gamma ^m$	mRNA decay rate
$\lambda ^p$	Protein translation rate
$\gamma ^p$	Protein decay rate
$\lambda _i^m$	mRNA production rate in i-th state
$\gamma _i^m$	mRNA decay rate in i-th state
$q_{ij}$	mRNA transition rate from state i to j
$\lambda _i^p$	Protein translation rate in i-th state
$\gamma _{\textrm{eff}}^m$	Effective mRNA decay rate
*K*	The number of mRNA states

The chemical reactions in (2) correspond to mRNA transcription and decay, transitions among multiple mRNA states, protein translation, and protein decay, respectively. Throughout this paper, we refer to model (2) as the *generalised two-stage model*, by which we mean that the model is treated as an extension of the classical two-stage model concerning the structuration of mRNA. We note that the (classical) two-stage model has been extended in this manner by the inclusion of an mRNA activation/inactivation loop recently [29]; however, here we generalise the results of [29] for a more comprehensive model. Additionally, we reanalyse published data on the influence of RNA stem loops on gene expression noise and explore the influence of kinetic rate parameters on predicted noise reduction ratios. From a biologically relevant standpoint, a similar model involving multiple mRNA states has recently been studied to quantify protein variability arising from mRNA-microRNA interactions [30].

In what follows, we present two specific examples which can be obtained from the structured/generalised model (2): the mRNA inactivation loop model and the multiphasic mRNA model. These models are given by the reactions

3 $$phi \mathop \rightleftharpoons \limits_{\gamma _1^m}^{{\lambda ^m}} {\text{mRNA}},{\text{mRNA}}\mathop \rightleftharpoons \limits_{{q_{21}}}^{{q_{12}}} {\text{imRNA}},{\text{imRNA}}\xrightarrow{{\gamma _2^m}}\phi ,\,\,{\text{mRNA}}\xrightarrow{{{\lambda ^p}}}{\text{mRNA}} + {\text{protein}},{\text{protein}}\xrightarrow{{{\gamma ^p}}}\phi ,$$

where the abbreviation $\textrm{imRNA}$ stands for an inactive mRNA molecule, and by

4 $$phi \xrightarrow{{{\lambda ^m}}}{\text{mRN}}{{\text{A}}_{\text{1}}}\xrightarrow{{K\gamma _{{\text{eff}}}^m}}{\text{mRN}}{{\text{A}}_{\text{2}}}\xrightarrow{{K\gamma _{{\text{eff}}}^m}} \cdots \xrightarrow{{K\gamma _{{\text{eff}}}^m}}{\text{mRN}}{{\text{A}}_{\text{K}}}\xrightarrow{{K\gamma _{{\text{eff}}}^m}}\phi ,{\text{mRN}}{{\text{A}}_{\text{i}}}\xrightarrow{{{\lambda ^p}}}{\text{mRN}}{{\text{A}}_{\text{i}}} + {\text{protein}},i = 1, \ldots ,K,{\text{protein}}\xrightarrow{{{\gamma ^p}}}\phi ,$$

respectively. Here, the reaction system (3) accounts for the activation/inactivation of an mRNA molecule modelled by involving a pair of reversible chemical reactions. In (4), an mRNA molecule is considered to move through its finite lifetime stages, which corresponds to the ageing of an mRNA. For a detailed discussion of these models, we refer the reader to Sections The mRNA inactivation loop model and Multiphasic mRNA lifetime and also the reference [29].

This paper is structured as follows. The core part of this study is given in Section Methods, where the generalised model is introduced in an in-depth analysis of a modelling framework. Specifically, in Section Model formulation, a brief review of the classical two-stage gene-expression model is given in stochastic settings; the underlying chemical master equation (CME) is transformed into a partial differential equation (PDE) for the generating function. Then, the main focus of this paper, which is the introduction of a generalization of the two-stage model, along with its corresponding CME and PDE, is presented. In Section Solution, a power series solution to the PDE is obtained. In Section Marginal distributions and moments, not only are the marginal mRNA and protein distributions obtained using the non-trivial analytical formula for the generating function, but the moments of the protein distributions are also determined by utilising factorial cumulants. The protein distribution is thereby recovered. Section Results pertains to data analysis, its interpretation, and summarises some of the key results of our mathematical analysis. The paper is concluded in Section Conclusions..

## Results

The motivation for our mathematical analysis stems from a recent experimental study [12] on the influence of RNA stem loops on gene expression noise. Stem loops appear when two palindromic sequences on the chain of nucleic acids align and form hydrogen bonds. The aligned palindromic sequences then form the “stem” and the nucleic acids in between form the “loop” of a stem loop. Another term is “hairpin loop” because of resemblance.

The authors of [12] constructed several variants of a gene encoding for a fluorescent reporter protein. Although the constructs encode for the same reporter protein, they differ in palindromic sequences in the untranslated region at the 5’ end of the gene (5’UTR). The formation of a stem loop interferes with translation; the higher the stability of a stem loop, the greater the interference; the lower the mean. The authors also show that this is associated with an increase in the coefficient of variation (CV).

**Fig. 1:**
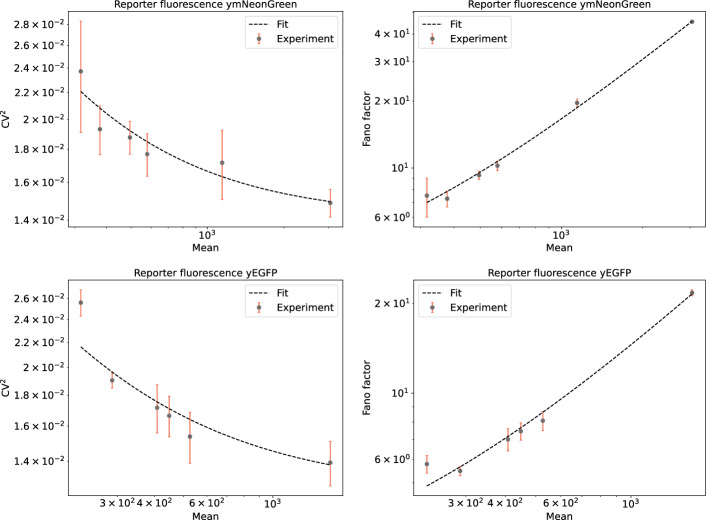
Dependence of protein noise on protein mean for different 5’UTR constructs. The yEGFP reporter (bottom) and the ymNeonGreen reporter (top) constructs are treated separately. The use of a log-log scale is adopted from [12]. The dots give the experimental values taken from [12] (see Table 2). Each dot is a result of multiple experiments, and the error bars indicate the standard deviation (SD). These were obtained from the standard deviation of the (nonsquared) coefficient of variation by Taylor formula: $SD_{CV^2} = 2 CV SD_{CV}$, $SD_F = \langle P \rangle SD_{CV^2}$. The dashed lines give the linear and hyperbolic dependence of the *F* and $CV^2$, respectively, which are predicted by the two-stage gene expression model (cf. (5)). The protein translation rate $\lambda ^p$ and the mRNA decay rate $\gamma ^m$ are being varied to change the mean levels. Note that the use of the log-log scale results in a slight curvature of the line (with a nonzero intercept)

Previous theoretical studies indicate that different noise metrics can lead to different interpretations of the effects of a particular mechanism on gene expression noise. The most common are the squared coefficient of variation and the Fano factor defined by

$$\text {CV}^2 = \frac{\langle P^2 \rangle - \langle P \rangle ^2}{\langle P \rangle ^2}, \quad \textrm{F} = \frac{\langle P^2 \rangle - \langle P \rangle ^2}{\langle P \rangle },$$

where *P* stands for the reporter protein and $\langle . \rangle$ are the averaging brackets. In Fig. 1, in addition to showing the dependence of the $\text {CV}^2$ on mean (thus reproducing Fig. 1 of [12]), we also show the dependence of $\textrm{F} = \langle P \rangle \text {CV}^2$ on the mean. Notably, decreasing the mean (which is associated with greater stem loop stability) decreases the Fano factor.

In order to explain the apparently contradictory interpretations, we fit the classical two-stage (transcription-translation) model (1) of gene expression [15, 24]. The model is described in full mathematical detail in Section Model formulation. For the purposes of the current section, we mention that it predicts that the stationary protein mean and Fano factor of the form

$$\langle P \rangle = \frac{\lambda ^m \lambda ^p}{\gamma ^m \gamma ^p}, \quad \textrm{F} = 1 + \frac{\lambda ^p}{\gamma ^m + \gamma ^p},$$

where $\lambda ^m$ is the mRNA production rate, $\lambda ^p$ is the protein translation rate, $\gamma ^m$ and $\gamma ^p$ are the decay rate constants of mRNA and protein species, respectively. We note that here in the expressions for the classical two-stage model, we omit the subscript *i* on mRNA in the generalised model (2) because there is only one mRNA state. Provided that the protein is more stable than the mRNA ($\gamma ^p \ll \gamma ^m$), we can simplify to

5 $$\begin{aligned} \textrm{F} = 1 + \frac{\lambda ^p}{\gamma ^m} = 1 + \frac{\gamma ^p \langle P \rangle }{\lambda ^m}, \quad \text {CV}^2 = \frac{\textrm{F}}{\langle P \rangle } = \frac{1}{\langle P \rangle } + \frac{\gamma ^p}{\lambda ^m}. \end{aligned}$$

Stem loops do not affect the transcription rate $\lambda ^m$ or the protein stability $\gamma ^p$, but they can affect the protein mean through translation rate $\lambda ^p$ and mRNA decay rate $\gamma ^m$. Thus, the two-stage model predicts an increasing linear dependence of the Fano factor, and a decreasing hyperbolic dependence of the $\text {CV}^2$, on the mean. In Fig. 1, the Fano factor data are fit by a straight line using simple linear regression. The slope of the regression line corresponds to the fraction $\lambda ^p/\gamma ^m$ in (5), which is calculated as 0.0141 and 0.0124 for the ymNeonGreen and yEGFP reporters, respectively. The regression coefficients are reused for the hyperbolic dependence of the $\text {CV}^2$. The fits seem to be satisfactory, leading us to attribute the changes in the noise to the decrease of mean rather than an active control of noise by the stem–loop mechanism. In the same Fig. 1, a reporter of gene expression, yeast-enhanced green fluorescent protein (yEGFP), and a monomeric protein, yellow mNeonGreen (ymNeonGreen), are used to obtain the experimental results.

**Table 2:** Protein mean and noise (CV) values for the yEGFP and the ymNeonGreen reporters obtained from [12]. The first column denotes distinct constructs that are of different stabilities. For instance, the L0 (P$_{\text {TEF}1}$) construct is driven by a strong promoter P$_{\text {TEF}1}$, generating a large abundance of protein molecules per cell, whereas P$_{\text {PAB}1}$ is a mid-range promoter. The hyphen symbol denotes undetermined values

	yEGFP		ymNeonGreen	
Construct	$\mu$	CV (%)	$\mu$	CV (%)
L0 (P$_{\text {TEF}1}$)	1560	11.8 ± 0.5	3050	12.2 ± 0.3
U	526	12.4 ± 0.6	–	–
M1Ug	408	13.1 ± 0.6	–	–
M3g	226	16.0 ± 0.4	–	–
G$_{10}$	448	12.9 ± 0.5	–	–
G$_{14}$	–	–	317	15.4 ± 1.5
M3Wn	–	–	1143	13.1 ± 0.8
M3n	–	–	579	13.3 ± 0.5
M3Un	–	–	377	13.9 ± 0.6
L0 (P$_{\text {PAB}1}$)	288	13.8 ± 0.2	495	13.7 ± 0.4

Let us address the question of noise control by stem–loop formation theoretically. For reasons of mathematical elegance, we will introduce a general model that extends the classical two-stage model (1) by multiple transcript states in Section Model formulation and provide a thorough analysis of the mRNA inactivation model (3) in Sections Solution-Marginal distributions and moments. Here we discuss the special case with two states, one of them translationally active (without a stem–loop), the other translationally inactive (with a stem–loop) (cf. Eq. (3)). This special case is analysed in Section The mRNA inactivation loop model. Importantly, we note that our results pertain to this special case; therefore, we drop the subscript *i* on mRNA species (cf. Eqs. (45) and (46)). Using standard methods, we derive that the mean is given by

$$\langle P \rangle = \frac{\lambda ^p \lambda ^m}{\gamma ^p \gamma _{\textrm{eff}}^m},$$

where

6 $$\begin{aligned} \gamma _{\textrm{eff}}^m = \gamma _1^m + \frac{q_{12} \gamma _2^m}{\gamma _2^m+q_{21}} \end{aligned}$$

gives an effective mRNA decay rate constant. The Fano factor satisfies

7 $$\begin{aligned} \textrm{F} = 1 + \frac{\lambda ^p}{\gamma ^p+\gamma _1^m+ \frac{q_{12}(\gamma ^p+\gamma _2^m)}{\gamma ^p+\gamma _2^m+q_{21}}}. \end{aligned}$$

The above equations give the steady-state protein mean and Fano factor as function of the model parameters (degradation rate constants $\gamma _1^m, \gamma _2^m, \gamma ^p$ of active/inactive mRNA and protein; inactivation/activation rate constants $q_{12}, q_{21}$; translation rate constant $\lambda ^p$). The formula for the mean implies, in particular, that making the stem–loop more stable (i.e. decreasing $q_{21}$) decreases the mean. The noise requires a more subtle analysis, which is given below.

In order to compare the protein noise in the current model to that exhibited by the classical two-stage model (without the inactivation–activation loop) we define the baseline Fano factor as

8 $$\begin{aligned} \textrm{F}_0 = 1 + \frac{\lambda ^p}{\gamma ^p + \gamma _{\textrm{eff}}^m} = 1 + \frac{\lambda ^p}{\gamma ^p + \gamma _1^m + \frac{q_{12} \gamma _2^m}{\gamma _2^m + q_{21}}}, \end{aligned}$$

which can be obtained from (7) by first setting $q_{12} = 0$ (no inactivation) and then replacing the mRNA decay rate $\gamma _1^m$ by its effective value (6). Adjusting the mRNA decay rate maintains the same species means in the baseline model like in the full model extended by the inactivation loop.

**Fig. 2:**
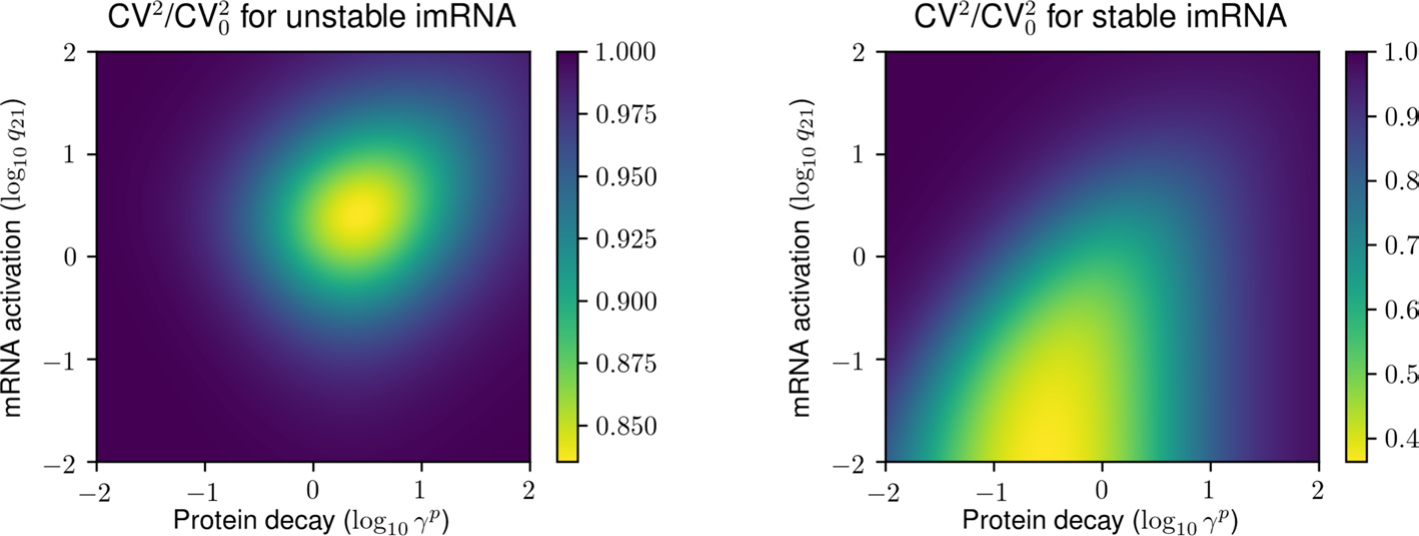
Fractional protein noise reduction by the mRNA inactivation loop as function of protein decay and mRNA activation rate constants. The colour of the heat map gives the protein noise (the squared coefficient of variation) in the two-stage model extended by the mRNA inactivation loop relative to the protein noise in a baseline two-stage model without the mRNA inactivation loop (adjusting the mRNA decay rate to obtain the same species means). The mRNA mean is set to $\langle M \rangle = 10$, and protein mean is $\langle P \rangle = 500$. The mRNA decay rate is set to $\gamma _1^m = 1$ without loss of generality; the inactive mRNA decay rate is either the same as that of active mRNA ($\gamma _2^m =1$; left panel) or set to zero ($\gamma _2^m = 0$; right panel). The inactivation rate constant is $q_{12} =3$

The protein variability formulae (7) and (8) can equivalently be expressed in terms of the squared coefficient of variation [31, 32] CV$^2 = \textrm{F}/\langle P \rangle$ and CV$_0^2 = \textrm{F}_0/\langle P \rangle$. We find that

9 $$\begin{aligned} \textrm{CV}^2&= \frac{1}{\langle P \rangle } + \frac{1}{\langle M \rangle } \frac{\gamma ^p}{\gamma ^p + \gamma _1^m +\frac{q_{12} (\gamma ^p + \gamma _2^m)}{\gamma ^p + \gamma _2^m +q_{21}}},\end{aligned}$$

10 $$\begin{aligned} \textrm{CV}^2_0&= \frac{1}{\langle P \rangle } + \frac{1}{\langle M \rangle } \frac{\gamma ^p}{\gamma ^p + \gamma _1^m +\frac{q_{12} \gamma _2^m}{\gamma _2^m +q_{21}}}, \end{aligned}$$

where $\langle M \rangle = \lambda ^m / \gamma _{\textrm{eff}}^m$ is the mean value of the activated mRNA.

Comparing (9) to (10), we see that $\textrm{CV}^2 < \textrm{CV}^2_0$, allowing us to conclude that the inclusion of the mRNA inactivation loop decreases protein noise. The key ingredient that distinguishes (9) from (10) is the Michaelis-Menten-type term in the denominator that involves the protein decay rate $\gamma ^p$, the mRNA activation rate $q_{21}$ and the inactive mRNA decay rate $\gamma _2^m$. Figure 2 explores the dependence of the fractional protein noise reduction $\textrm{CV}^2/\textrm{CV}^2_0$ on these parameters. Without loss of generality, the active mRNA decay rate is set to one, and two plausible alternatives are considered for the decay of the inactive mRNA molecule: one, imRNA is unstable, decaying with the same rate as mRNA (left panel of Fig. 2); two, imRNA is stable, i.e. protected from degradation, and does not decay (right panel of Fig. 2). We observe qualitatively different noise reduction patterns for the two alternatives. For an unstable imRNA, there is an optimal combination of mRNA activation and protein decay rate constants that minimise the protein noise (Fig. 2, left panel). We observe that the optimal values are both greater than the unit value of the mRNA decay rate. This requirement runs counter to the biological evidence that proteins are typically more stable than mRNAs. The maximal reduction of noise is moderate (around 15% for the chosen parameter set). For a stable imRNA, the optimum is approached by making the mRNA activation as slow as possible (Fig. 2, right panel). The optimal value of protein decay rate constant is then less than that of the active mRNA, and the maximal reduction of noise is more pronounced (around 60% for the chosen parameter set).

Our analysis goes beyond the first and second moments (means and variances). In particular, for the mRNA inactivation loop model (3), we show that its steady state distribution is generated by Taylor-expanding the explicit function

$$G({x_1},{x_2},z) = \exp \left( {\frac{{{\lambda ^m}{\lambda ^p}}}{{\gamma _{eff}^m{\gamma ^p}}}\int\limits_1^z {2{F_2}\left( {\begin{array}{*{20}{c}} {1,1 + \tau } \\ {1 + {r_1},1 + {r_2}} \end{array};\frac{{{\lambda ^p}}}{{{\gamma ^p}}}(s - 1)} \right)ds + \frac{{{\lambda ^m}({x_1} - 1)}}{{\gamma _{eff}^m}}2{F_2}\left( {\begin{array}{*{20}{c}} {1,1 + \tau } \\ {1 + {r_1},1 + {r_2}} \end{array};\frac{{{\lambda ^p}}}{{{\gamma ^p}}}(z - 1)} \right) + \frac{{{q_{12}}{\lambda ^m}({x_2} - 1)}}{{\gamma _{eff}^m(\gamma _2^m + {q_{21}})}}2{F_2}\left( {\begin{array}{*{20}{c}} {1,\tau } \\ {1 + {r_1},1 + {r_2}} \end{array};\frac{{{\lambda ^p}}}{{{\gamma ^p}}}(z - 1)} \right).{\text{ }}} } \right)$$

The conjugate variables $x_1$, $x_2$, and *z* correspond to mRNA, imRNA, and protein species, respectively. Parameters $r_1$, $r_2$, and $\tau$ are parameter groupings defined by (53) and (55), respectively. The symbol $_{2}F_{2}$ stands for

$$\begin{aligned} _p F_q\left( \begin{array}{l} a_1, \ldots , a_p \\ b_1, \ldots , b_q \end{array}; \tilde{z}\right) =\sum _{n=0}^{\infty } \frac{\left( a_1\right) _n \ldots \left( a_p\right) _n}{\left( b_1\right) _n \ldots \left( b_q\right) _n} \frac{\tilde{z}^n}{n!} \end{aligned}$$

which is the generalised hypergeometric function [33].

Our mathematical analysis thus provides a complete characterisation of the steady state distribution in the mRNA inactivation model in particular (as well as the generalised model in general), and extends the generating function result previously given for the two stage model in [18].

## Conclusions

In this paper, we formulated and analysed a structuration/generalisation of the two-stage gene expression model in terms of having multiple mRNA states. Unlike the classical two-stage model, the generalised model considers multiple mRNA states, among which mRNA molecules are assumed to be transitioning at constant rates. Additionally, we demonstrated that the generalised model can be used to capture the dynamics of simpler models such as the inactivation loop model and the multiphasic mRNA model, which were analysed in detail as a particular interest of this paper.

We first introduced the corresponding chemical reaction system describing the generalised model and its mathematical description given by the CME. Then, we focused on seeking a solution to the corresponding PDE, which is obtained by transforming the CME using the generating function approach. A suitable ansatz was employed for converting the PDE to a system of ODEs. Subsequently, using the power series method, we sought a solution to the ODE system, which is then expressed in matrix form as a system of recurrence equations. We recovered the generating function of the stationary distribution of mRNA and protein amounts by means of the coefficients of power series, which are obtained by solving the recurrence relations under the initial conditions.

Furthermore, the sought-after solution was then used to characterise the marginal protein and mRNA distributions. To determine the protein distribution, we used the factorial moments, which are calculated from the factorial cumulants. Additionally, we demonstrated that the mRNA distributions are Poissonian. Obtaining a Poisson distribution is evident for any monomolecular chemical reaction system [34]; therefore, we derived the protein mean and Fano factor and thus expressed it in terms of the first two factorial moments. We then provided two different examples to which the generalised model and its results can be applied.

The first example concerns the inactivation loop model. We demonstrated that integrating the mRNA inactivation loop into the classical two-stage framework for gene expression results in reduced values of protein noise. Nevertheless, we note that certain conditions on the parameter rates must be met to obtain a significant protein noise reduction. These constraints take different forms depending on the interaction between the mRNA form and the mRNA degradation pathway. The first option is that the formation of the inactivation loop does not interfere with degradation, meaning that the inactive form degrades with the same rate constant as the active form. The second option is that the formation of the loop interferes with degradation so that its instantaneous degradation rate becomes zero. In both cases, protein stability must be optimally chosen to maximise noise reduction; the protein can be neither too stable nor too unstable. However, if inactive mRNAs are subject to degradation, noise reduction is optimised for relatively low protein stabilities, whereas if inactive mRNAs are protected from degradation, noise reduction is optimised for more realistic, larger values of protein stabilities. For inactive mRNAs that do not degrade, optimal noise reduction requires low mRNA activation rates, whereas relatively fast rates of activation optimise noise reduction if inactive mRNAs degrade. Generally, the stability of the inactive mRNA form sustains greater reductions of protein noise for wider and more realistic parameter values. Overall, the noise analysis suggests that the mRNA inactivation loop may play a role in controlling gene expression noise, while also highlighting the limitations of its effect. It is worth noting that one can also compare the protein variance between the extended and canonical two-stage models using the mRNA autocovariance function [35]. The approach taken in this work has an additional advantage that we present a notably non-trivial distribution for protein, which is expressed in terms of the generalised hypergeometric series and is employed to obtain a recursive expression for the protein probability mass function.

As a second example, by making suitable parameter choices in the generalised model, we presented the multiphasic model in which an mRNA molecule is assumed to be transitioning through its lifetime stages. The solution obtained for the generalised model and the associated matrices (e.g., the transition matrix) were used to determine the first two moments of mRNA distributions, which allowed us to calculate the Fano factor for the multiphasic model.

We provided a biological example of the formation of RNA stem loops and performed data analysis to explain the influence of stem-loop structure on gene expression noise. Specifically, we based our extensive mathematical analysis on the two standard noise metrics: the CV$^2$ and the Fano factor. By doing so, our calculations allowed us to conclude that noise in gene expression can be reduced if stem loops restrict translation.

In summary, the paper provides a systematic mathematical analysis for protein–mRNA interactions in a structured gene expression model. We believe that the model and its results can be used in understanding the dynamics of underlying biochemical processes.

## Methods

### Model formulation

For the two-stage gene expression model (1), the probability $p_{m,n}(t)$ of observing *m* mRNA and *n* protein molecules at time *t* satisfies the CME

11 $$\begin{aligned} \begin{aligned} \frac{\textrm{d}}{\textrm{d} t} p_{m,n} =&\lambda ^m (p_{m-1,n} - p_{m,n}) + \gamma ^m ((m+1) p_{m+1,n} - m p_{m,n} ) \\&+ \lambda ^p m (p_{m,n-1} - p_{m,n}) + \gamma ^p ((n+1) p_{m,n+1} - n p_{m,n}), \end{aligned} \end{aligned}$$

subject to initial condition

$$\begin{aligned} p_{m,n}(0) = \delta _{m,m_0} \delta _{n,n_0}, \end{aligned}$$

where $\delta _{i,j}$ represents the Kronecker delta symbol, which is one if $i=j$ and zero otherwise; $m_0$ and $n_0$ are the initial mRNA and protein amounts, respectively.

Our aim is to obtain a PDE rather than working with the CME (11). To this end, we introduce the probability generating function defined by

12 $$\begin{aligned} G(x,y,t) = \sum _{m} \sum _{n} x^m y^n p_{m,n}(t). \end{aligned}$$

Multiplying the CME (11) by the factor $x^m y^n$ and summing over all *m* and *n*, and using (12), we arrive at the generating function which satisfies the linear first-order PDE

13 $$\begin{aligned} \frac{\partial G}{\partial t} = (\gamma ^m (1-x) + \lambda ^p x (y-1) ) \frac{\partial G}{\partial x} + \gamma ^p (1-y) \frac{\partial G}{\partial y} + \lambda ^m (x-1) G. \end{aligned}$$

Equation (13) has been used in [24] to derive mRNA and protein moments; it has been solved at steady state in [18]. Here we shall derive and study a generalisation of (13).

Without loss of generality, for the generalised model (2), the probability $P(\textbf{m}, n, t)$ of observing $m_1$ mRNA copies in state 1, $m_2$ mRNA copies in state 2, and so on, at given time *t* satisfies the following CME,

14 $$\begin{aligned} \begin{aligned} \frac{\text {d} P(\textbf{m},n, t)}{\textrm{dt}} =&\sum _{i=1}^{K} \left( \lambda _i^m (\mathbb {E}^{-1}_i-1)P + \gamma _i^m (\mathbb {E}_i-1) m_i P + \sum _{j=1}^{K} q_{ij} (\mathbb {E}_i\mathbb {E}^{-1}_j-1)\right. \\&\times m_i P + \left. \lambda _i^p (\mathbb {E}^{-1}_{K+1}-1) m_i P \right) + \gamma ^p (\mathbb {E}_{K+1}-1) n P, \end{aligned} \end{aligned}$$

where $$\textbf{m} = \begin{bmatrix} m_1&m_2&m_3&\ldots&m_K \end{bmatrix}$$ is a vector of species copy numbers. Note that the step operator [36] $\mathbb {E}_i$ in (14) is in the variable $m_i$, whereas $\mathbb {E}_{K+1}$ is in the variable *n*; $\mathbb {E}_i \mathbb {E}^{-1}_j-1 = 0$ for $i=j$.

The multivariate probability generating function is given by

15 $$\begin{aligned} G(\textbf{x}, y,t) = \sum _{m_1}\cdots \sum _{m_K}\sum _{n}P(\textbf{m},n,t) x_1^{m_1} x_2^{m_2}\cdots x_K^{m_K} y^n, \end{aligned}$$

where $$\textbf{x} = \begin{bmatrix} x_1&x_2&x_3&\ldots&x_K \end{bmatrix}$$. Multiplying (14) by $x_1^{m_1} x_2^{m_2}\ldots x_K^{m_K} y^n$ and summing over all $m_1, m_2, \ldots , m_K, n$, and employing (15), we arrive at the PDE

16 $$\begin{aligned} \begin{aligned} \frac{\partial G(\textbf{x},y,t)}{\partial t} =&\sum _{i=1}^{K} \left( \lambda _i^m (x_i-1) G + \gamma _i^m (1 - x_i) \frac{\partial G}{\partial {x_i}} + \sum _{j=1}^{K} q_{ij} (x_j - x_i) \frac{\partial G}{\partial {x_i}} \right. \\&+ \left. \lambda _i^p (y-1) x_i \frac{\partial G}{\partial {x_i}} \phantom {\sum _{i=1}^{K}} \right) + \gamma ^p (1-y) \frac{\partial G}{\partial {y}}. \end{aligned} \end{aligned}$$

Note that the step operators $\mathbb {E}_i^{\pm 1}$ in (14) coincide with the variables $x_i^{\mp 1}$ while the copy number of species $m_i$ correspond to the terms $x_i \partial _{x_i}$ in (16) for the generating function. In the next section, we will seek a solution to the PDE (16).

### Solution

In this section, we shall provide a step-by-step breakdown of our solution method for solving the PDE (16). We are interested in the steady state; therefore, we set the time derivative in (16) to zero and rearrange the resulting equation to obtain

17 $$\sum\limits_{i = 1}^K {\left( {\lambda _i^m({x_i} - 1)G + \left( {\gamma _i^m(1 - {x_i}) + \sum\limits_{j = 1}^K {{q_{ij}}} ({x_j} - {x_i}) + \lambda _i^p(y - 1){x_i}} \right)\frac{{\partial G}}{{\partial {x_i}}}} \right)} + {\gamma ^p}(1 - y)\frac{{\partial G}}{{\partial y}} = 0$$

for the time-independent generating function $G(\textbf{x},y)$ of the stationary distribution. The probability normalisation condition translates to $G(1,\ldots ,1) = 1$. Changing the variables according to

18 $$\begin{aligned} x_i = 1 + u_i, \quad y = 1 + v, \quad G = \exp (\varphi ) \end{aligned}$$

allows us to transform (17) into

19 $$\begin{aligned} \sum _{i=1}^{K} \left( \lambda _i^m u_i + \left( \lambda _i^p v(1+u_i) - \gamma _i^m u_i + \sum _{j=1}^{K} q_{ij} (u_j - u_i) \right) \frac{\partial \varphi }{\partial u_i} \right) = \gamma ^p v \frac{\partial \varphi }{\partial v}, \end{aligned}$$

which is subject to the normalisation condition

20 $$\begin{aligned} \varphi (\textbf{0}) = 0. \end{aligned}$$

Below, we focus on seeking a solution to (19)–(20) using a suitable ansatz.

Let us first consider that the solution is of the form

21 $$\begin{aligned} \varphi (u_1, u_2, u_3, \ldots ,u_K, v) = \varphi _0(v) + u_1 \varphi _1(v) + \ldots + u_K \varphi _K(v) . \end{aligned}$$

With this in mind, we obtain from (21) that

22 $$\begin{aligned} \frac{\partial \varphi }{\partial u_i} = \varphi _i(v), \quad \frac{\partial \varphi }{\partial v} = \varphi _0^{\prime }(v) + u_1 \varphi _1^{\prime }(v) + \ldots + u_K \varphi _K^{\prime }(v). \end{aligned}$$

Inserting the partial derivatives (22) into (19), we get

23 $$\begin{aligned} \begin{aligned} \sum _{i=1}^{K} \left( \lambda _i^m u_i + \left( \lambda _i^p v (1+u_i)- \gamma _i^m u_i + \sum _{j=1}^{K} q_{ij} (u_j - u_i) \right) \varphi _i - \gamma ^p v u_i \varphi _i^\prime \right) \\ = \gamma ^p v \varphi _0^\prime . \end{aligned} \end{aligned}$$

Equation (23) can be rewritten as

24 $$\left( {{\gamma ^p}\varphi _0^\prime - \sum\limits_{i = 1}^K {\lambda _i^p} {\varphi _i}} \right)\nu + \sum\limits_{i = 1}^K {\left( {{\gamma ^p}v\varphi _i^\prime + \left( {\gamma _i^m - \lambda _i^p\nu + \sum\limits_{j = 1}^K {{q_{ij}}} } \right){\varphi _i} - \sum\limits_{j = 1}^K {{q_{ji}}} {\varphi _j} - \lambda _i^m} \right)} {u_i} = 0.$$

In order that (24) hold, we must necessarily have

25 $$\begin{aligned}&\sum _{i=1}^{K} \lambda _i^p \varphi _i - \gamma ^p \varphi _0^\prime = 0, \end{aligned}$$

26 $$\begin{aligned}&\gamma ^p v \varphi _i^\prime + \left( \gamma _i^m - \lambda _i^p v + \sum _{j=1}^{K} q_{ij} \right) \varphi _i - \sum _{j=1}^{K} q_{ji} \varphi _j = \lambda _i^m. \end{aligned}$$

Thus far, we have converted the system of PDEs (17) into the system of ODEs (25)–(26). Next, we provide a detailed explanation of solving this system using the power series method.

Let us assume that the functions $\varphi _0$ and $\varphi _i$ are of the power series form, i.e.,

27 $$\begin{aligned} \varphi _0(v) = \sum _{n=0}^{\infty } a_n v^n, \quad \varphi _i(v) = \sum _{n=0}^{\infty } b_n^{(i)} v^n \end{aligned}$$

for $i \in \{1,\ldots ,K \}$. Differentiating (27) term by term we get

28 $$\begin{aligned} \varphi ^{\prime }_0(v) = \sum _{n=1}^{\infty } n a_n v^{n-1}, \quad \varphi ^{\prime }_i(v) = \sum _{n=1}^{\infty } n b_n^{(i)} v^{n-1}. \end{aligned}$$

Inserting (27) and (28) into (26), and collecting same powers of *v*, we obtain the following system of recurrence relations

29 $$\begin{aligned} \left( \gamma _i^m + \sum _{j=1}^{K}q_{ij} + n \gamma ^p \right) b_n^{(i)} - \sum _{j=1}^{K} q_{ji} b_n^{(j)} = \lambda _i^p b_{n-1}^{(i)} \end{aligned}$$

for the coefficients $b_n^{(i)}$, where $i=1,\ldots ,K$. For the sake of simplicity, equations (29) can be rewritten in matrix form as

30 $$\begin{aligned} (\mathbf {A-Q^\top } + n \gamma ^p \textbf{I}) X_n = \textbf{B} X_{n-1}, \quad n \ge 1, \end{aligned}$$

where **I** is the identity matrix and the vector $X_n$ is defined as

$$\begin{aligned} X_n = \begin{bmatrix} b_n^{(1)},&b_n^{(2)},&b_n^{(3)},&\ldots ,&b_n^{(K)} \end{bmatrix}^\top . \end{aligned}$$

In (30), **A** is a $K \times K$ matrix defined by

31 $$\begin{aligned} \textbf{A}_{ij} := {\left\{ \begin{array}{ll} \gamma _i^m & \text { for } i=j,\\ 0 & \text { for } i \ne j, \end{array}\right. } \end{aligned}$$

$\textbf{Q}$ is a $K \times K$ matrix defined by

32 $$\begin{aligned} \textbf{Q}_{ij} := {\left\{ \begin{array}{ll} - \sum \limits _{k \ne i} q_{ik} & \text { for } i=j,\\ q_{ij} & \text { for } i \ne j, \end{array}\right. } \end{aligned}$$

and $\textbf{B}$ is a $K \times K$ matrix defined by

33 $$\begin{aligned} \textbf{B}_{ij} := {\left\{ \begin{array}{ll} \lambda _i^p & \text { for } i=j,\\ 0 & \text { for } i \ne j. \end{array}\right. } \end{aligned}$$

In order to solve the recurrence relations (30) initial conditions are needed. These can be obtained from (26) by setting $v=0$ for each $i \in \{1,2,\ldots ,K \}$. The resulting system of linear equations is given in matrix form as

34 $$\begin{aligned} \mathbf {(A-Q^\top )} X_0 = C, \end{aligned}$$

where C is a column vector defined as $$C = \begin{bmatrix} \lambda _1^m&\lambda _2^m&\ldots&\lambda _K^m \end{bmatrix}^\top$$.

Solving the system of algebraic equations (30) under the initial conditions (34) yields the terms of $b_{n}^{(i)}$; the sequence $a_n$ can be obtained by substituting (27) and (28) into (25) and collecting same powers of *v*. By doing so, we get

35 $$\begin{aligned} a_{n} = \frac{1}{n \gamma ^p} \sum _{i=1}^{K} \lambda _i^p b_{n-1}^{(i)}, \quad n \ge 1. \end{aligned}$$

Note that the normalisation condition (20) implies that $a_0 = \varphi _0(0) = \varphi (\textbf{0}) = 0$. Having found the sequences $a_n$ and $b_n^{(i)}$, we combine (21) and (27) to obtain

36 $$\begin{aligned} \varphi (u,v) = \sum _{n=1}^{\infty } a_n u^n + \sum _{i=1}^{K} v_i \sum _{n=0}^{\infty } b_n^{(i)} u^n. \end{aligned}$$

We return to the original variables in (36) via (18) to obtain the generating function of the stationary distribution of mRNA and protein amounts, which is given by

37 $$\begin{aligned} G(\textbf{x},y) = \exp \left( \sum _{n=1}^{\infty } a_n (y-1)^n + \sum _{i=1}^{K} (x_i - 1) \sum _{n=0}^{\infty } b_n^{(i)} (y-1)^n \right) . \end{aligned}$$

Equation (37) provides the sought-after steady-state solution to the PDE (16) and will be used in the following section.

### Marginal distributions and moments

In this section, we use the analytical formula for the generating function (37) to obtain marginal mRNA distributions. We determine the moments of the protein distribution by way of the factorial cumulants, which allow us to recover the protein distribution. Additionally, we derive the protein Fano factor (variance-to-mean ratio) and express it in terms of the first two factorial moments.

*Marginal mRNA distributions* In the generating function (37), if we take $y=1$, then we obtain the marginal mRNA distributions as

38 $$\begin{aligned} G^m(\textbf{x}) = G(\textbf{x}, 1) = \exp \left( \sum _{i=1}^{K} b_0^{(i)} (x_i - 1) \right) = \prod _{i=1}^{K} \exp \left( b_0^{(i)} (x_i - 1) \right) , \end{aligned}$$

from which we conclude that the steady state mRNA distributions are independent Poissons with means

$$\begin{aligned} \langle m_i \rangle = b_0^{(i)}. \end{aligned}$$

*Marginal protein distribution* Likewise, by inserting $x_i = 1$ ($i=1,\ldots ,K$) into (37), we can recover the generating function of the marginal protein distribution

39 $$\begin{aligned} G(y) = G(\textbf{1}, y)= \exp \left( \sum _{n=1}^{\infty } a_n (y-1)^n \right) , \end{aligned}$$

where $\textbf{1}$ is a *K*-dimensional row vector of ones.

Next, we determine the moments of the protein distributions. The factorial (combinatorial) moments $h_n$ are obtained by expanding the generating function into a power series around $y=1$:

$$\begin{aligned} G(y) = \sum _{n=0}^{\infty } h_n (y-1)^n. \end{aligned}$$

We aim to calculate the factorial moments $h_n$ by way of the factorial cumulants $a_n$. To that end, we first differentiate (39) to obtain

40 $$\begin{aligned} \textrm{D}G(y) = G(y) \textrm{D} \ln G(y), \end{aligned}$$

where $\textrm{D}$ denotes the differential operator $\textrm{d}/\textrm{d}y$. Then, taking the $(n-1)$th derivative of (40), we get

$$\begin{aligned} \textrm{D}^n G(y) = \sum _{i=0}^{n-1} \left( {\begin{array}{c}n-1\\ i\end{array}}\right) \textrm{D}^i G(y) \textrm{D}^{n-i} \ln G(y), \end{aligned}$$

which can be recast as

41 $$\begin{aligned} \frac{\textrm{D}^n G(y)}{n!} = \sum _{i=0}^{n-1} \left( 1 - \frac{i}{n} \right) \frac{\textrm{D}^i G(y)}{i!} \frac{\textrm{D}^{n-i} (\ln G(y))}{(n-i)!}. \end{aligned}$$

Evaluating (41) at $y=1$ gives the factorial moments of the protein distribution

42 $$\begin{aligned} h_n = \sum _{i=0}^{n-1} \left( 1 - \frac{i}{n} \right) a_{n-i} h_i, \quad \text { for } n \ge 1, \end{aligned}$$

where $h_0 = 1$. The terms of $h_n$ can be recursively obtained by inserting (35) into (42). Subsequently, by employing the recurrence method proposed in [37], we recover the protein distribution

$$\begin{aligned} p(n) = \sum _{j=1}^{\infty } \frac{(j+1)_n}{n!} h_{n+j}(-1)^j, \end{aligned}$$

where $(x)_n$, *n* being a nonnegative integer, denotes the rising factorial or namely Pochhammer symbol.

*Moments* Clearly, the mRNA distributions in (38) are Poissonian. Therefore, mRNA Fano factor is equal to 1. The protein mean and Fano factor can be derived from the factorial moments (42). The first two factorial moments are given by

43 $$\begin{aligned} \langle n \rangle = h_1 = a_1 \quad \text {and} \quad \langle n(n-1) \rangle = 2h_2 = 2 a_2 + a_1^2, \end{aligned}$$

respectively. The Fano factor,

44 $$\begin{aligned} \textrm{F} = \frac{\langle n^2 \rangle }{\langle n \rangle } - \langle n \rangle = \frac{\langle n(n-1) \rangle }{\langle n \rangle } + 1 - \langle n \rangle = \frac{2a_2}{a_1} + 1, \end{aligned}$$

is thus expressed in terms of the first two factorial cumulants $a_1$ and $a_2$.

### The mRNA inactivation loop model

In this section, we present a particular example of the generalised model (2), which we refer to as the *inactivation loop model*, whose reaction scheme is given by (3). Specifically, we provide an explicit representation of the stationary solution using the cumulants. Furthermore, we calculate the steady-state protein Fano factor and express it as a function of the model parameters. Let us note that a possible biological scenario that can implement this model is by a regulatory RNA that temporarily blocks mRNA function [38].

The inactivation loop model (3) can be readily obtained from the generalised model (2) by taking $K=2$, which accounts for only two mRNA states denoting the active mRNA state $m_1$ and the inactive mRNA state $m_2$. In what follows, we assume that a newly produced mRNA is active, i.e. that the transcription rate satisfies

45 $$\begin{aligned} \lambda _i^m = \lambda ^m \delta _{i,1}, \quad \text {for } i=1,2. \end{aligned}$$

Additionally, we assume that proteins are translated only from an active mRNA, so that we have

46 $$\begin{aligned} \lambda _i^p = \lambda ^p \delta _{i,1}, \quad \text {for } i=1,2, \end{aligned}$$

for the translation rate. Here, $\delta _{i,j}$ denotes the Kronecker delta symbol. *Cumulants* We aim to recover expressions for the inactivation loop model from the generalised model. The system of algebraic equations for this model follows from (34), taking the form of

$$\begin{aligned} (\gamma _1^m + q_{12}) b_0^{(1)} - q_{21} b_0^{(2)}&= \lambda ^m, \\ (\gamma _2^m + q_{21}) b_0^{(2)} - q_{12} b_0^{(1)}&= 0, \end{aligned}$$

from which we recover

47 $$\begin{aligned} b_0^{(1)} = \frac{\lambda ^m (\gamma _2^m + q_{21})}{(\gamma _1^m + q_{12})(\gamma _2^m + q_{21}) - q_{12}q_{21}}. \end{aligned}$$

Combining (47) with (39) we find

$$\begin{aligned} \langle m_1 \rangle = \frac{\lambda ^m}{\gamma _{\textrm{eff}}^m}, \end{aligned}$$

where

48 $$\begin{aligned} \gamma _{\textrm{eff}}^m = \gamma _1^m + \frac{q_{12} \gamma _2^m}{\gamma _2^m+q_{21}} \end{aligned}$$

is the effective rate of mRNA decay. The recurrence relations (30) read

49 $$\begin{aligned}&(\gamma _1^m + q_{12} + n \gamma ^p) b_n^{(1)} - \lambda ^p b_{n-1}^{(1)} - q_{21} b_n^{(2)} = 0, \end{aligned}$$

50 $$\begin{aligned}&(\gamma _2^m + q_{21} + n \gamma ^p) b_n^{(2)} - q_{12} b_n^{(1)} = 0, \end{aligned}$$

for $n \ge 1$. Solving the algebraic system (49)–(50) in $b_n^{(1)}$ yields

51 $$\begin{aligned} b_n^{(1)} = \frac{\lambda ^p (\gamma _2^m + q_{21} + n \gamma ^p)}{{\gamma ^p}^2 n^2 + \gamma ^p(\gamma _2^m + \gamma _1^m +q_{21}+q_{12}) n + \gamma _2^m \gamma _1^m +\gamma _1^m q_{21} + \gamma _2^m q_{12}} b_{n-1}^{(1)}, \end{aligned}$$

which is a recursive expression whose first term (i.e. zeroth) is given by (47).

*Explicit representation* The recursive formula (51) can further be simplified by factorising its denominator as

52 $$\begin{aligned} b_n^{(1)} = \lambda ^p \frac{\gamma _2^m+ q_{21} + n \gamma ^p}{{\gamma ^p}^2 (n+r_1)(n+r_2)} b_{n-1}^{(1)} \quad \text {for } n \ge 1, \end{aligned}$$

where

53 $$\begin{aligned} r_{1,2} = \frac{\gamma _1^m + q_{12} + \gamma _2^m + q_{21}\pm \sqrt{(\gamma _2^m + q_{21} - \gamma _1^m - q_{12})^2 + 4 q_{21} q_{12}}}{2\gamma ^p} \end{aligned}$$

are the opposite numbers to the roots of the quadratic in the denominator of (51). The sequence (52) can be rewritten as

54 $$\begin{aligned} b_n^{(1)} = \frac{\lambda ^m \left( 1+ \tau \right) _n }{\gamma _{\textrm{eff}}^m (1+r_1)_n (1+r_2)_n } \left( \frac{\lambda ^p}{\gamma ^p}\right) ^n , \quad {n\ge 1}, \end{aligned}$$

where

55 $$\begin{aligned} \tau = \frac{\gamma _2^m + q_{21}}{\gamma ^p}, \end{aligned}$$

and $(x)_n$ represents the rising factorial. Thus, $a_n$ can be obtained from (35) as

56 $$\begin{aligned} a_{n} = \frac{\lambda ^p}{n \gamma ^p} b_{n-1}^{(1)}, \quad n \ge 1. \end{aligned}$$

Inserting (54) into (56) gives

57 $$\begin{aligned} a_n = \frac{\lambda ^m r_1 r_2}{\gamma _{\textrm{eff}}^m \tau } \frac{\left( \tau \right) _n}{n (r_1)_n (r_2)_n}\left( \frac{\lambda ^p}{\gamma ^p}\right) ^n, \quad n \ge 1, \end{aligned}$$

and, similarly, inserting (54) into (50) gives

58 $$\begin{aligned} b_n^{(2)} = \frac{q_{12} \lambda ^m \left( \tau \right) _n}{\gamma _{\textrm{eff}}^m(\gamma _2^m + q_{21}) (1+r_1)_n (1+r_2)_n} \left( \frac{\lambda ^p}{\gamma ^p}\right) ^n, \quad n \ge 1. \end{aligned}$$

Substituting (54), (57), and (58) into (37), we obtain an explicit representation of the stationary solution

$$\begin{gathered} G\left( {x_{1} ,x_{2} ,z} \right) = \exp \left( {\frac{{\lambda ^{m} \lambda ^{p} }}{{\gamma _{{eff}}^{m} \gamma ^{p} }}\int\limits_{1}^{z} {{}_{2}F_{2} \left( {\begin{array}{*{20}c} {1,1 + \tau } \\ {1 + r_{1} ,1 + r_{2} } \\ \end{array} ;\frac{{\lambda ^{p} }}{{\gamma ^{p} }}(s - 1)} \right)ds} } \right. \hfill \\ \quad \quad \quad \quad \quad \quad \quad + \frac{{\lambda ^{m} \left( {x_{1} - 1} \right)}}{{\gamma _{{eff}}^{m} }}{}_{2}F_{2} \left( {\begin{array}{*{20}c} {1,1 + \tau } \\ {1 + r_{1} ,1 + r_{2} } \\ \end{array} ;\frac{{\lambda ^{p} }}{{\gamma ^{p} }}(z - 1)} \right) \hfill \\ \quad \quad \quad \quad \quad \quad \quad \left. { + \frac{{q_{{12}} \lambda ^{m} \left( {x_{2} - 1} \right)}}{{\gamma _{{eff}}^{m} \left( {\gamma _{2}^{m} + q_{{21}} } \right)}}{}_{2}F_{2} \left( {\begin{array}{*{20}c} {1,\tau } \\ {1 + r_{1} ,1 + r_{2} } \\ \end{array} ;\frac{{\lambda ^{p} }}{{\gamma ^{p} }}(z - 1)} \right)} \right), \hfill \\ \end{gathered}$$

where

$$_p F_q\left( \begin{array}{c} a_1, \ldots , a_p \\ b_1, \ldots , b_q, \ldots \end{array}; \tilde{z}\right) =\sum _{n=0}^{\infty } \frac{\left( a_1\right) _n \ldots \left( a_p\right) _n}{\left( b_1\right) _n \ldots \left( b_q\right) _n} \frac{\tilde{z}^n}{n!}$$

is the generalised hypergeometric function [33]. Furthermore, combining (47) and (56) yields an equivalent expression

$$\langle n \rangle = \frac{\lambda ^p \lambda ^m (\gamma _2^m + q_{21})}{\gamma ^p((\gamma _1^m + q_{12})(\gamma _2^m + q_{21}) - q_{12}q_{21})} = \frac{\lambda ^p \lambda ^m}{\gamma ^p \gamma _{\textrm{eff}}^m}$$

for the protein mean given by (43) in terms of the model parameters. Likewise, substituting (56) and (51) into (44) and simplifying gives

59 $$\begin{aligned} \textrm{F} = 1 + \frac{b_n^{(1)}}{b_n^{(0)}} = 1 + \frac{\lambda ^p}{\gamma ^p+\gamma _1^m+ \frac{q_{12}(\gamma ^p+\gamma _2^m)}{\gamma ^p+\gamma _2^m+q_{21}}} \end{aligned}$$

for the steady-state protein Fano factor as function of the model parameters.

### Multiphasic mRNA lifetime

In this section, we consider that mRNA molecules posses $K > 2$ stages of their lifetime, where the transition rates correspond to the ageing of an mRNA molecule. The chemical reaction system for this multiphasic model was given in (4). We note that kinetic proof reading cascades can be an interesting application of our multiphasic model [39].

By (4), there are *K* stages of an mRNA’s molecule lifetime, each of which lasts $1 / K \gamma _{\textrm{eff}}^m$ on average. The total mRNA lifetime is then $1 / \gamma _{\textrm{eff}}^m$; $\gamma _{\textrm{eff}}^m$ is thereby interpreted as the effective mRNA decay rate. The multiphasic mRNA decay in *K* steps leads to an Erlang-distributed lifetime with mean $1 / \gamma _{\textrm{eff}}^m$ and variance $1 / (\gamma _{\textrm{eff}}^m)^2$, whereas the lifetime distribution is exponential in the standard model (1).

The multiphasic model (4) can be obtained by making the following choices in the general model statement (2):

$$\lambda _{i}^{m} = \left\{ {\begin{array}{*{20}l} {\lambda ^{m} } \hfill & {{\text{ for }}i = 1,} \hfill \\ 0 \hfill & {{\text{ for }}i \ne 1,} \hfill \\ \end{array} } \right.$$

and

$$\begin{aligned} \gamma _i^m = {\left\{ \begin{array}{ll} K \gamma _{\textrm{eff}}^m & \text { if } i=K,\\ 0 & \text { otherwise}. \end{array}\right. } \end{aligned}$$

The transition matrix $\textbf{Q}$ (32) for the multiphasic model takes the form of

60 $$\begin{aligned} \textbf{Q} = K \gamma _{\textrm{eff}}^m \begin{pmatrix} -1 & 1 & & & \\ & -1 & 1 & & \\ & & \ddots & \ddots & \\ & & & -1 & 1\\& & & & 0 \end{pmatrix}, \end{aligned}$$

and the matrix $\textbf{A}$ (31) is given by

61 $$\begin{aligned} \textbf{A} = K \gamma _{\textrm{eff}}^m \begin{pmatrix} 0 & & & & \\ & 0 & & & \\ & & \ddots & & \\ & & & 0 & \\ & & & & 1 \end{pmatrix}. \end{aligned}$$

Inserting (61) and (60) into (34), we obtain the system of recurrence equations

62 $$\begin{aligned} K \gamma _{\textrm{eff}}^m \begin{pmatrix} 1 & & & & \\ -1 & 1 & & & \\ & -1 & \ddots & & \\ & & \ddots & 1 & \\ & & & -1 & 1 \end{pmatrix} \begin{pmatrix} b_0^{(1)}\\ b_0^{(2)}\\ \vdots \\ b_0^{(i)}\\ \vdots \\ b_0^{(K)} \end{pmatrix} = \begin{pmatrix} \lambda ^m\\ 0\\ \vdots \\ 0\\ \vdots \\ 0 \end{pmatrix}, \end{aligned}$$

from which, upon taking the *i*-th row of (62) and solving the recursive equations

$$\begin{aligned} - K \gamma _{\textrm{eff}}^m b_0^{(i-1)} + K \gamma _{\textrm{eff}}^m b_0^{(i)} = 0, \quad \text {for } 2 \le i \le K, \end{aligned}$$

where $b_0^{(1)} = \lambda ^m / K \gamma _{\textrm{eff}}^m$, we recover

63 $$\begin{aligned} b_0^{(i)} = \frac{\lambda ^m}{K \gamma _{\textrm{eff}}^m}. \end{aligned}$$

Formula (63) gives the mean of mRNA molecule in the *i*-th state of its lifetime. Note that the matrix $\textbf{B}$ (33) takes the form of $\textbf{B} = \lambda ^p \textbf{I}$, where $\textbf{I}$ is the identity matrix.

Having found the first moments (i.e. means) (63), we then determine the second moments. Taking $n=1$ in (30), we have

64 $$\begin{aligned} \begin{pmatrix} K \gamma _{\textrm{eff}}^m + \gamma ^p & & & & \\ -K \gamma _{\textrm{eff}}^m & K \gamma _{\textrm{eff}}^m + \gamma ^p & & & \\ & -K \gamma _{\textrm{eff}}^m & \ddots & & \\ & & \ddots & K \gamma _{\textrm{eff}}^m + \gamma ^p & \\ & & & -K \gamma _{\textrm{eff}}^m & K \gamma _{\textrm{eff}}^m + \gamma ^p \end{pmatrix} \begin{pmatrix} b_1^{(1)}\\ b_1^{(2)}\\ \vdots \\ b_1^{(i)}\\ \vdots \\ b_1^{(K)} \end{pmatrix} = \frac{\lambda ^p \lambda ^m}{K \gamma _{\textrm{eff}}^m} \begin{pmatrix} 1\\ 1\\ \vdots \\ 1\\ \vdots \\ 1 \end{pmatrix}, \end{aligned}$$

from which we obtain the first term of the sequence $b_1^{(i)}$ as

65 $$\begin{aligned} b_1^{(1)} := u = \frac{\lambda ^p \lambda ^m}{K \gamma _{\textrm{eff}}^m (K \gamma _{\textrm{eff}}^m + \gamma ^p)}. \end{aligned}$$

Equation (64) implies that

$$\begin{aligned} - K \gamma _{\textrm{eff}}^m b_1^{(i-1)} + (K \gamma _{\textrm{eff}}^m + \gamma ^p) b_1^{(i)} = \frac{\lambda ^p \lambda ^m}{K \gamma _{\textrm{eff}}^m}, \quad \text {for } 2 \le i \le K, \end{aligned}$$

which can equivalently be rewritten as

66 $$\begin{aligned} b_1^{(i)} = u + v b_1^{(i-1)}, \quad 2 \le i \le K, \end{aligned}$$

where we set

67 $$\begin{aligned} v = \frac{K \gamma _{\textrm{eff}}^m}{K \gamma _{\textrm{eff}}^m + \gamma ^p} \end{aligned}$$

for simplicity. Combining (66) and (65), we obtain

68 $$\begin{aligned} b_1^{(i)} = \frac{u}{1-v} + v^{i-1} \left( u - \frac{u}{1-v}\right) , \quad 1 \le i \le K, \end{aligned}$$

from which all the elements of $b_1^{(i)}$ (thereby the second moments) can be iteratively obtained. It is worth noting that one can derive higher moments using formula (30), but we limit our study to the first two moments.

Next, we focus on calculating the first two terms of the sequence $a_n$ (35). Setting $n=1,2$ in (35) and inserting (63) and (68) into the resulting equations, respectively, we get

69 $$\begin{aligned} a_1 = \frac{\lambda ^p \lambda ^m}{\gamma ^p \gamma _{\textrm{eff}}^m} \quad \text { and} \quad a_2 = \frac{\lambda ^p u\left( K+v \left( -1 - K + v^K \right) \right) }{2 \gamma ^p(1-v)^2}. \end{aligned}$$

Having found the first two terms of $a_n$, we are now ready to calculate the Fano factor. Inserting (69) into (44), and substituting (65) and (67) into the resulting expression yields

$$\begin{aligned} \mathrm {F_{m}} = 1 + \frac{\lambda ^p}{\gamma ^p} \left( 1 + \frac{\gamma _{\textrm{eff}}^m}{\gamma ^p} \left( -1 + \left( \frac{K \gamma _{\textrm{eff}}^m}{K \gamma _{\textrm{eff}}^m + \gamma ^p} \right) ^K \right) \right) , \end{aligned}$$

where $\mathrm {F_{m}}$ stands for the multiphasic Fano factor.

## Data Availability

Not applicable.

## Abbreviations

mRNA: Messenger RNA
CME: Chemical master equation
PDE: Partial differential equation
ODE: Ordinary differential equation
imRNA: Inactive mRNA
UTR: Untranslated region
CV: Coefficient of variation
SD: Standard deviation
yEGFP: Yeast-enhanced green fluorescent protein
ymNeonGreen: Yellow monomeric fluorescent protein
